# Wearable Sensor-Based Gait Analysis for Age and Gender Estimation

**DOI:** 10.3390/s20082424

**Published:** 2020-04-24

**Authors:** Md Atiqur Rahman Ahad, Thanh Trung Ngo, Anindya Das Antar, Masud Ahmed, Tahera Hossain, Daigo Muramatsu, Yasushi Makihara, Sozo Inoue, Yasushi Yagi

**Affiliations:** 1 Department of Media Intelligent, Osaka University, Ibaraki 567-0047, Japan; trung@am.sanken.osaka-u.ac.jp (T.T.N.); muramatsu@am.sanken.osaka-u.ac.jp (D.M.); makihara@am.sanken.osaka-u.ac.jp (Y.M.); yagi@sanken.osaka-u.ac.jp (Y.Y.); 2 Department of Electrical and Electronic Engineering, University of Dhaka, Dhaka 1000, Bangladesh; idno.s123@gmail.com; 3 Electrical Engineering & Computer Science, University of Michigan, Ann Arbor, MI 48109, USA; anindyadasantar@gmail.com; 4 Graduate School of Life Science and Systems Engineering, Kyushu Institute of Technology, Kitakyushu 804-8550, Japan; taheramoni@gmail.com (T.H.); sozo@brain.kyutech.ac.jp (S.I.)

**Keywords:** gait, recognition, wearable sensor, age estimation, gender, smartphone

## Abstract

Wearable sensor-based systems and devices have been expanded in different application domains, especially in the healthcare arena. Automatic age and gender estimation has several important applications. Gait has been demonstrated as a profound motion cue for various applications. A gait-based age and gender estimation challenge was launched in the 12th IAPR International Conference on Biometrics (ICB), 2019. In this competition, 18 teams initially registered from 14 countries. The goal of this challenge was to find some smart approaches to deal with age and gender estimation from sensor-based gait data. For this purpose, we employed a large wearable sensor-based gait dataset, which has 745 subjects (357 females and 388 males), from 2 to 78 years old in the training dataset; and 58 subjects (19 females and 39 males) in the test dataset. It has several walking patterns. The gait data sequences were collected from three IMUZ sensors, which were placed on waist-belt or at the top of a backpack. There were 67 solutions from ten teams—for age and gender estimation. This paper extensively analyzes the methods and achieved-results from various approaches. Based on analysis, we found that deep learning-based solutions lead the competitions compared with conventional handcrafted methods. We found that the best result achieved 24.23% prediction error for gender estimation, and 5.39 mean absolute error for age estimation by employing angle embedded gait dynamic image and temporal convolution network.

## 1. Introduction

The number of elderly people is increasing globally, especially in Japan, South Korea, New Zealand, and some European countries. Overall, the aging society is expanding and hence, more new challenges are around to explore. As per the World Health Organization (WHO), by 2020, the number of people aged 60 years or more will be more than the number of children younger than 5 years [1]. According to the current estimation, the number of 60 years or older people will be 2 billion by 2050 [1]. According to this trend, after three decades, countries like China, Iran, Chile, Russian Federation will have proportionally more elderly people. Therefore, it is a demanding task to explore research areas and automation—so that we can support elderly people in smarter manners, improve healthcare facilities, and enhance social welfare, while considering the socio-economic scenarios of the respective nations. Automated healthcare—whether sensor-based or vision-based—have become essential for the future [2,3,4,5,6]. Wearable devices are extensively explored in various applications of healthcare and elderly support [7,8,9]. There are ample of opportunities to explore various wearable sensors in the domain of activities of daily living (ADL), gait analysis, exercise, heart rate monitoring, respiration rate, and other vital information analysis. Among various wearable and other sensors, smartphones and smartwatches become very popular due to the ease of use, availability, relatively cost-effective as people use phones a lot, and the data collection is easier from the in-built accelerometer, gyroscope, or other sensors [10,11,12]. Inertial measurement unit (IMU) sensors are extensively explored in various research works and systems. Several survey works are done by [13,14], where the authors highlighted the accelerometer-based human gait analysis and various clinical applications to monitor as well as diagnose several diseases.

Gait recognition and gait-based systems have been studied over the last two decades. With the advent of deep learning approaches, the research activities on gait have expanded much more than the past, especially in the computer vision domain. Gait-based healthcare monitoring and assessment can be very much helpful for the future. As gait is the most dominant human movement and is one of the most important biometrics—it can be useful to understand any physical as well as medical conditions and well-being.

A healthcare system needs to know the age as well as the gender of a user so that the system can be more realistic and assistive. Gait patterns vary with the variations of age along with gender. Wearable sensors can be exploited to decipher the age or gender of a subject. Based on the age group or gender, a system can more accurately provide healthcare support. Understanding age or gender has other applications in various fields. However, estimations of age and gender are not much explored using wearable sensors [9,15]. The existing approaches are limited to small datasets and with limited scopes. Therefore, to mitigate these constraints, we introduced a challenge in a top-ranked conference (*The 12th IAPR International Conference on Biometrics* (ICB), Greece, 2019), where a large scale dataset is offered for the global participants. The competition was based on two OU-ISIR inertial datasets that have gait data of 745 subjects for training and an additional dataset for testing. The dataset is developed by a good number of participants—ranging from various age groups as well as by both sexes. The competition was very successful with a number of good participants from all over the world.

**Figure 1:**
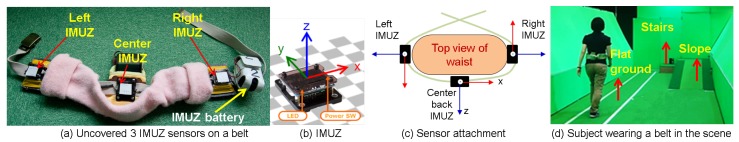
Setup of the sensor-based human gait data capturing system: (**a**) Waist-belt (uncovered) having three IMUZ sensors; (**b**) three axes of a typical IMUZ sensor; (**c**) Sensors’ attachment at left, right, and center-back position; and (**d**) Real data collection image, where a subject is wearing a belt, and flat ground, stairs and slope are highlighted in the environment. (This Figure was previously published in [17] as Figure 8. Hence, it is reprinted from [17], Copyright (2015), with permission from Elsevier).

In this paper, we highlight the importance of gait, summarize various approaches related to gait, and age and gender estimations. Then we introduce the training and testing datasets and outline the competition along with the evaluation approach. Afterwards, we summarize the features and methods, which are explored by the participants on this dataset. Results and discussions are appended before concluding the paper with some future work guidelines. A shorter version of this challenge was published in [16]. In this feature article, we rigorously enhance the previous paper [16] with more extended presentations, new related work (there was no related work in the earlier version), enhanced descriptions of datasets, enriched evaluation methods and features (enhanced by more than 12 times, in terms of texts), and detailed results and analysis. Without the references, the number of texts in this paper is 4.5 times that of the earlier version. We also added some future work guidelines based on the existing challenges. We added more than five times the number of new references (mostly from journal papers), along with a few new tables. All the tables are upgraded with finer information and analysis. We rigorously analyzed the methods and articulated the preprocessing and feature extraction approaches for gender classification and age prediction for the algorithms. In a similar fashion, various classification approaches are dealt with. We added seven completely new figures/graphs to enrich the presentations and all illustrations are upgraded with more informative data and approaches. We extract the top algorithms for age estimation and gender prediction, irrespective of any team to decipher the profound methodologies for each challenging arena. Only the Figure 1 is reprinted from [17], Copyright (2015), with permission from Elsevier. Therefore, this version of the paper has highly engraved enriched information, analysis, and discussions.

## 2. Related Works

Elderly citizens need more care as fall, injury, various diseases, walking disorders, dementia, etc. become more common to them. For example, about 75% of injury-producing falls on steps occur for the people of 65 years or older [18]. Another study says that about one-third of the population over 65 years of age have at least one fall down event [19]. In fact, several studies claim that by knowing and studying the gait characteristics, we can differentiate a person who might fall or not, predict future fall cases for a subject, or risk issues [20].

Gaits are important to know any developmental progress during some diseases or after some surgeries. Some important health and medical-related terminologies that can be addressed by gait characteristics are patients with cerebral palsy, cerebellar ataxia, knee arthroplasty, hip arthroplasty, Charcot-Marie-Tooth disease, idiopathic arthritis, idiopathic Parkinson’s disease, atypical Parkinson’s disease, bipolar disorder, dementia, Pick’s disease, idiopathic normal pressure hydrocephalus, ankle osteoarthritis, Alzheimer’s disease, postural instability, and gait difficulty, and to improve the life-quality of patients and elderly people who are suffering due to gait-related or walking pattern-related problems [13,21,22].

In healthcare applications, several works explored gait or walking patterns. For example, in the work by [23], older adults showed more gait variability (especially, spatio-temporal variability) during Two-Minute Walk Tests (2MWT), as well as, Six-Minute Walk Tests (6MWT). In this research, 31 participants (16 young adults and 15 older adults) used six wireless inertial sensors on each foot till waist. They predicted that the 2MWT was preferable to research and clinical gait assessment. 36 patients (18 females and 18 males) who had total hip arthroplasty (THA) were studied with inertial sensors. Gait parameters were computed for further analysis. Ahmed et al. [24] studied some physiological developments from smartphone sensor-based gait data. They collected data from 66 subjects in different age groups. Accelerometer and gyroscope were explored for gait signals from 60 patients having Parkinson’s disease (24 females and 36 males) [25]. They computed several spatio-temporal gait parameters (e.g., gait velocity, stride length, maximum toe clearance, and cadence), then employed a machine learning algorithm for evaluation.

For age estimation based on gait analysis, a number of works are accomplished with various findings. For example, any sudden turn or stop while walking can provide kinematic information that can differentiate age and gender [26]. Gait speed slows down with age and this issue has been studied by [27,28,29,30,31]. Ref. [30] performed gait characteristics study with 118 adults (58 females and 60 males)—ages ranging from 20 to 79 years. They explored the functionality of age and gender on gait. There were four age groups: 20 to 39 years, 40 to 59 years, 60 to 69 years, and 70 to 79 years. For kinematic analysis, they used nine markers on the lower part of the body. According to their findings, with age, the gait pattern was changed, mainly due to the decreasing muscle strength. However, ref. [32] extensively studied the impact of muscle weakness and gait. Their finding is that human gait is very sensitive to the weakness of plantar-flexors and hip abductors. In this research, the subjects were only six (three females and three males) and only 15 years or older.

Reference [28] studied on the comfortable and maximum walking speed of adults on a range of ages 20 to 79 years old. The paper by [29] investigated adults who were aged from 20 to 82 years, having almost equally-distributed genders (58 females and 50 males). According to their study, they recommend that age-related variations are mainly due to the step-length pattern and relatively, less related to aging. Some gender-specific differences were noticed by their research, though these are not conclusive. These are several early-stage works where age or gender issues are incorporated for the investigation of gait or walking patterns for young adults to elderly people.

In another research, a collection of 1860 healthy subjects (941 females and 919 males; ages ranging from 5 to 100 years) were explored for gait data collection [33]. They used a mobile inertial sensor-based system called RehaWatch system, for gait data collection. Each system had three accelerometers and three gyroscopes. They created eight age groups: 5 to 10, 10 to 15, 15 to 20, 20 to 30, 30 to 40, 40 to 50, 50 to 60, 60 to 70, and 70 to 100. From their study, they identified a significant non-linear relationship between age and gait parameters.

Age and gender issues are combined by a number of works. For example, age- and gender-related tests were conducted by [34] on elderly people. They did the following four common tests that are clinically done: Six-minute Walk Test (6MWT), Berg Balance Scale (BBS), Timed Up and Go Test (TUG), and gait speeds (comfortable gait speed and fast gait speed). These were done on 96 elderly adults (age range: 61 to 89 years), and three age cohorts were created as: 60 to 69, 70 to 79, and 80 to 89 years. Their preliminary result depicted that age-related data should be considered by physical therapists for any analysis. In addition, for both males and females, there was a realistic trend of decline for the clinical tests they did on the adults.

Effect of age, gender, and speed of walking were investigated for adults from 20 to 60 years by [35]. They found that gender difference in the performance of gait was significant, while age issues were varied for both genders on different parameters. On the other hand, the work by [36] investigated gait characteristics, and motor and sensory abilities of 122 older adults (both male (32) and female (90) subjects, having about 70 year of average age). They claimed that the aging process affects both males and females differently. Therefore, they suggested that to improve the gait and balance of older people, gender difference issues should be considered.

The work by [15] attached IMUs in four locations of the body of 26 subjects (14 females and 12 males) to estimate gender, age, and height. They analyzed accelerations and angular velocities, and computed 50 features in that time and frequency domains. In another work by [37], similar accelerations and angular velocities are computed from 86 subjects (37 females and 49 males). Similar to [15], they computed 50 features for age estimation. In this work, they created six age groups: 10 to 19, 20 to 29, 30 to 39, 40 to 49, 50 to 59, and 60+ year. Based on the datasets of the competition, Ref. [38] introduced several strategies for gender and age estimation based on various approaches. The best results are achieved by combining CNN and Angle Embedded Gait Dynamic Image (AE-GDI).

In another recent work by [39], gait performance was studied by inertial sensors. They engaged 378 adults having a mean age of 71 years (210 females and 168 males). Inertial sensors were attached to the lower trunk and heel. Accelerations, angular velocity and walking time were computed for further analysis. They introduced a score called Comprehensive Gait Assessment using InerTial Sensor score (C-GAITS score). Effect of treadmill walking on upper trunk and gait are studied in [40] where they engaged eight subjects. Mun et al. [21] introduced a CNN-based model to analyze gait estimation from various foot parameters. They employed multiple inertial sensors as well as a commercialized motion capture system to evaluate on 42 subjects. They introduced the system where foot features can be important predictors of individual gait. Usually, gait-related studies are accomplished by normal sensors or IMUs or video and rarely, commercialized motion capture systems were explored. Apart from walking gait, running gait patterns were studied using wearable sensors and a Random Forest-based model [41].

Smartphone sensors, force plates, electromyography (EMG), goniometer, sensing fabric, floor sensors, foot pressure insoles, etc. are also explored for these studies [13,42,43]. In most of the cases, tri-axial accelerometers are employed. In most of the works, one to two sensors are used though even 6/7 sensors are attached in different parts of the bodies by some methods. Apart from accelerometer, IMU, gyroscope, various sensing devices, video cameras (RGB or depth cameras) are also explored by some researchers for gait-based age or gender estimation. One of the earliest works is [44], where the authors explored the then large dataset. It had 1728 subjects, ages ranging from 2 to 94 years. Prior to this work, other datasets had a much lesser number of subjects (e.g., 122 subjects in USF dataset [45], or 170 subjects as mentioned in [44]). They implemented the Gaussian process regression approach for gait-based age estimation. USF dataset [45] is a smaller dataset having 122 subjects (37 females and 85 males, age range: 19 to 59). This dataset has age information. Ref. [46] has exploited label encoding and multi-label-guided subspace method on this dataset for age estimation. In another work by [47], gender recognition was done on their dataset called Advanced Digital Sciences Center-Arbitrary Walking Directions (ADSCAWD). They proposed a sparse reconstruction based metric learning method to extract gait features.

Later, video-based large datasets were explored for age or gender estimation. For example, ref. [48] proposed a method for age estimation and experimented on the OU-ISIR Gait Database, Large Population Dataset with Age (OULP-Age). This dataset had more than 60,000 participants and age ranges from 2 to 90 years old. In their group-dependent framework based on manifold, they achieved excellent results. In another work exploring multi-stage CNN by [49], video-based gait-based age was estimated. In this paper, they introduced CNN-based models for gender estimation method, age-group estimation, and age regression. They also explored the OULP-Age dataset for their evaluation and achieved a satisfactory result. There are age and gender prediction approach from face images using Residual networks of Residual networks (RoR) [50]. In another domain, speech signals are explored for age and gender classification [51].

In the above discussions, we mentioned various gait parameters or features that are explored. Here, we list them: step width, toe off angle, step length, step irregularity, gait cycle time, walking speed or time, lateral foot position, acceleration amplitude variability, cadence (number of steps per minute in steps/minute), cycle frequency, double support duration, foot symmetry, gait irregularity or variability, step asymmetry, stance duration, swing duration, walk ratio, and so on. Walking time, acceleration data in three axes are more widely computed along with angular velocity. However, the importance of each feature or parameter are varied from one work to another, as well as, from one application to another.

However, it is difficult to generalize from these above research works, because the number of participants is usually not high, the male-female ratio is not balanced in almost all cases, the age ranges are also varied in different cases and these are not benchmark datasets. Most of the adults are usually healthy older adults [36]. Moreover, most of the cases, the gait data are analyzed from small groups of subjects [33]. Apart from these points, almost none of these datasets are explored by a good number of research groups. Moreover, there are some conflicting results on the effect of walking speed on gait characteristics [20,34]. Therefore, it becomes difficult to generalize some findings for different or larger settings. Nevertheless, these findings are fruitful for further assessment and provide meaningful insights to take more actions. In order to mitigate some of the above constraints, we arranged the challenge based on a large dataset, having a huge number of participants. The dataset has a large variations in age distributions, as well as, almost equal distributions of both genders. Apart from these, we have a good number of algorithms tested on this large dataset—so that we can get more insights and methods that are more suitable for age and gender estimations based on sensor-based gait data.

## 3. Description of the Dataset

The training dataset was distributed at the beginning of the competition. However, the unlabeled test dataset was shared to the participants after the registration deadline. The datasets are designed from tri-axial inertial motion sensors. The training dataset consists of the following two sets:

(a) OU-ISIR Gait Database, Inertial Sensor Dataset [52].
(b) OU-ISIR Gait Database, Similar Action Inertial Dataset [17].

A part of the unlabeled test dataset is similar to the above two sets. The other subset was orchestrated on a flat ground. The location of sensors were attached in a backpack and the orientations of the sensors were varied.

### 3.1. Gait Capture System

The following sections present the gait capturing system. We split the presentation into a training dataset and a test dataset. In this setup, we exploited 3 inertial measurement unit (IMU) sensors named as IMUZ sensors (from ZMP Inc.) [53] to capture gait signals. Each IMUZ consists of a triaxial accelerometer and a triaxial gyroscope. The IMUZ dynamic ranges were at ±4 [g] and ±500 [deg/s] for capturing gait signals. These three sensors captured data at the frame rate of 100 Hz. This is the maximum frame rate for these sensors. These sensors transferred data to a computer through a Bluetooth connection. In terms of attachment on the body, we mounted these on a waist belt of the waist of each subject. Figure 1a,d depict the arrangement and on-body location of the sensors. In order to avoid any direct body-contact, the waist belt was covered by a soft cushion. It also provided a safety-net for the sensors. The three sensors are located at the left, one at the right, and the other one at center-back of the belt. The three sensors were fixed to the belt at different orientations (i.e., $90^{{}^{\circ}}$ for center-left, center-right pairs, and $180^{{}^{\circ}}$ for left-right pair of sensors).

#### 3.1.1. Training Dataset

**Figure 2:**
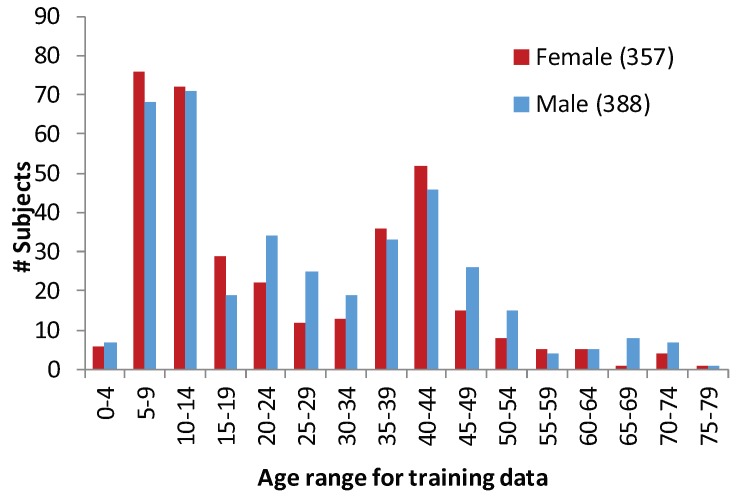
Distribution of subjects in training dataset—by age group and gender. The histogram demonstrates a non-uniform distribution of age groups though the distributions of both sexes are almost equally distributed.

Gait data of 745 visitors were collected for five days in an exhibition in Japan. The advantages of the dataset included a large variation of age (i.e., from 2 to 78 years) and equal gender balance (i.e., 388 males and 357 females, the average age is about 26 years). Each visitor walked in and out of the designated data capture booth only once, see Figure 1d. Informed consent to permit the use of these data for research purposes was taken from each participant. Distribution of the number of training subjects by age groups and genders are presented in Figure 2. We can note that the young children from 5 to 14 years for both sexes had the highest number of participants. There were very few participants in the age groups of 0–4 and more than 55 years old. This non-uniformity in terms of age groups is a constraint of this dataset. However, this issue was deemed a challenge as well. Though there were 16 age groups in the training set, we will find later that a few consecutive age groups were merged where there were less participants.

**Figure 3:**
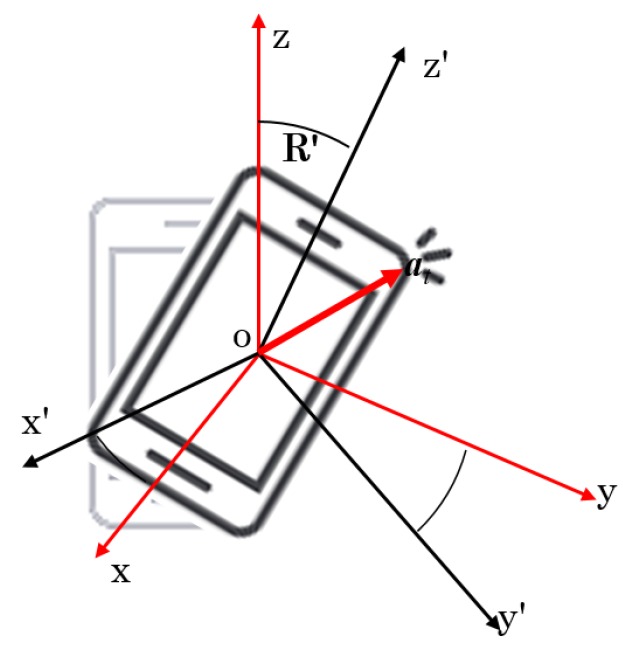
An example of sensor orientation inconsistency: within and among subjects.

Note that there are multiple challenges to handle in terms of sensor location inconsistency, and sensor orientation inconsistency (within and among subjects) (Figure 3 and Figure 1c). Moreover, the test sequence has the presence of noises. Therefore, these issues should be handled by the participants. Sensor orientation inconsistency is one of the core challenging issues in sensor-based human activity or gait recognition. There are a few approaches to make sensor-orientation invariant. Noise or missing data in the data sequence pose difficulties unless a method is proposed robustly.

We captured signal sequences from the triaxial accelerometer and triaxial gyroscope in each IMUZ sensor. Therefore, the captured data became 6D data. We collected five gait activities as: level, up-slope walk, down-slope walk, step up, and step down. Data only for *level*, *slope upward*, and *slope downward* walk was extracted for each subject. For the *level* walk, the dataset had two sequences (for entering and exiting the path) per subject. However, one sequence was extracted for the *slope upward* or *slope downward* walking patterns. There were four labels for data as: ID, Age, Gender, and Activity. These labels are published for the training dataset.

Simple preprocessing steps were done to strip any invalid data. The extracted data had unequal distributions per subject. Therefore, the training dataset contains three subsets with different variations. The number of subjects per age group is the most important factor. Hence, we created the first subset having the maximum number of subjects [52]. The second subset was maximized with the variations of sensor type and location, and ground condition. The issue related to a limited number of subjects is rationalized through these variations [52]. Finally, the third subset was created and maximized by having the variation of the ground condition [17].

#### 3.1.2. Test Dataset

The test dataset is different from the training dataset in terms of challenges and location of the sensors in one subset. The number of subjects was 58 (19 females and 39 males—mostly having younger people). One subset of the test dataset was captured in the same environment of the training dataset. These data were not published in the OU-ISIR gait dataset, inertial sensor dataset [52]. In this subset, there are 47 subjects, out of 58 subjects. These are taken from three inertial sensors, as in the case of the training dataset, in the same experimental environment. Unlike the training set, the test dataset has not published any labels.

**Figure 4:**
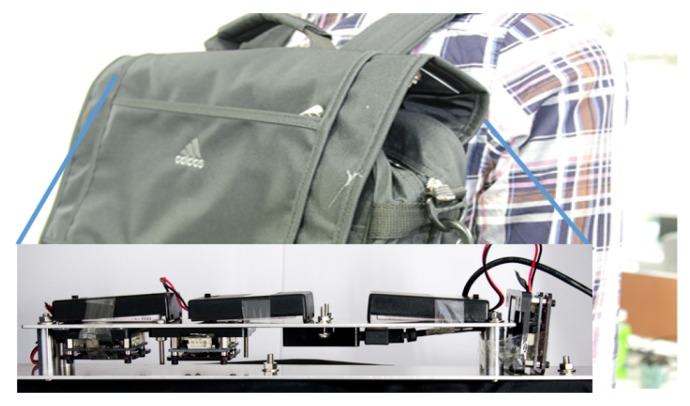
An example of three IMUZ sensors in the backpack for the test dataset. The sensors are attached to the top of the backpack.

**Figure 5:**
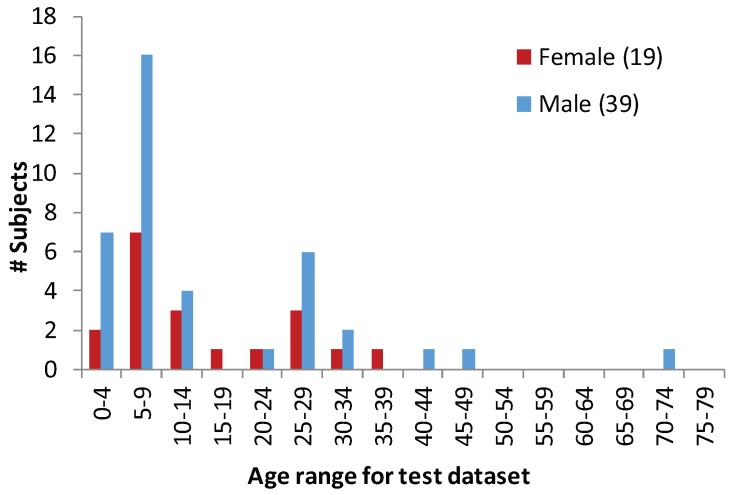
Distribution of subjects in test dataset—by age group and gender. The histogram demonstrates a much non-uniform distribution of age groups and gender than the training dataset.

**Figure 6:**
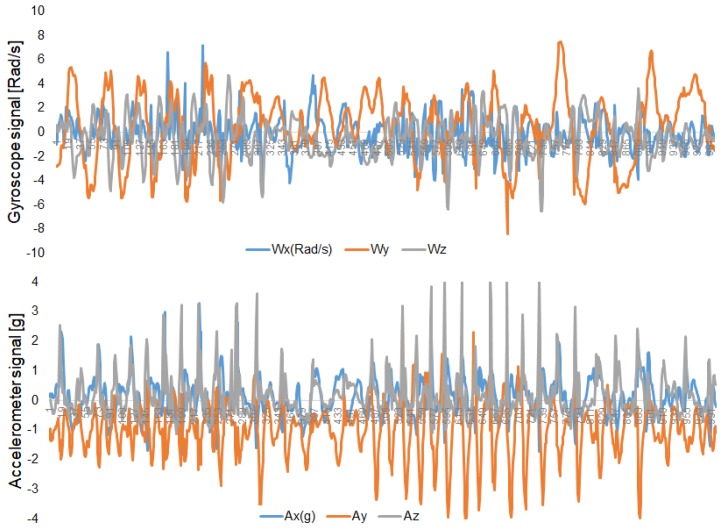
Examples of test signal sequences for gyroscope data, and accelerometer data.

The other subset of the test dataset was captured having the sensors in a different location—on a backpack as shown in Figure 4. These sensors were firmly attached to the backpack at three different orientations: i.e., $90^{{}^{\circ}}$ for center-left, left-right, and center-right pairs. This subset was created from 11 subjects with known age and gender, and two captured days per subject. This dataset was extracted from the dataset in [54], without any added load on the backpack. Through these processes, with different sensor-orientations and locations, and having different captured days, we finally created 194 test data sequences (58 subjects had 2 to 6 sequences). The distributions of gender and age for test dataset are shown in Figure 5. Figure 6 depicts an example of a test signal.

**Figure 7:**
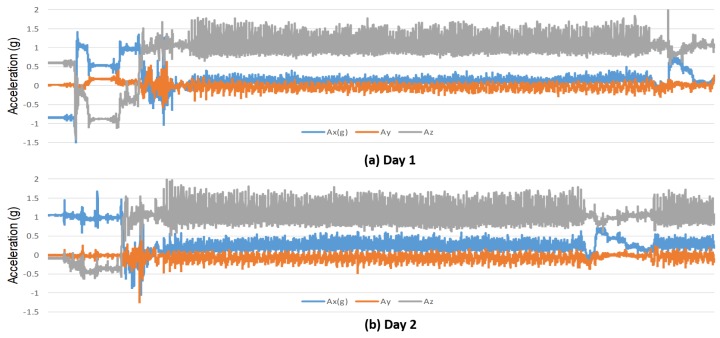
Examples of accelerometer data that appear only in testing.

The gender and age predictions were difficult because of the sensor orientations associated with each signal sequence are not disclosed. Apart from that, the sensor locations in the backpack are different from the waist. Figure 7 shows the accelerometer data for two days, which appeared only during testing. Subjects were requested to sign informed-consent to approve their data to explore for research purposes.

## 4. Employed Algorithms

As shown in Table 1, 18 teams from 14 countries did their registrations for the competition. They were from institutes, universities or companies from Bangladesh, Belgium, China, France, German, Italy, Japan, Norway, Pakistan, Singapore, Spain, Taiwan, the UK, and the USA. Finally, ten teams could finish and submit the evaluation results. Except for two teams, other teams submitted multiple solutions, comprising a total of sixty-seven algorithms for age and gender estimation. In the following sections, we denote ‘algorithm’ as ‘A’ (e.g., ‘Algorithm 1’ is ‘Alg.1’). There are 32 algorithms for gender estimation and 35 algorithms for age estimation. These are highlighted in Table 2.

### 4.1. Explored Features and Approaches

The datasets had accelerometer and gyroscope data. The submitted 67 algorithms or solutions did some preprocessing steps, feature vector extraction, and classification on these signal sequences. In the preprocessing step, few methods smartly handled the sensor orientation variation issue between the training and the test datasets. Table 3 demonstrates that four teams (i.e., T3, T6, T7, and T8) explored this issue.

Some algorithms of T3, T4, T7, and T9 used raw signals for further computation. T1 explored Fourier transformation, T7’s one solution (A2) used Short Time Fourier transformation approach. This solution computed Hidden Markov Models (HMMs), Gaussian Mixture Model (GMM), and a Universal Background Model (UBM) [56]. UBM is trained by using the Baum-Welch recursion approach on gait signals. Afterwards, an Eigen-gait projection matrix is computed using a probabilistic principal component analysis [57] for GMMs and HMMs. The Eigen-gait coefficients from the matrix are used for age or gender prediction.

T2, T9, and T10 exploited several statistical features, which are dominantly used in different sensor-based human activity recognition methods. Some of the employed statistical features are mean, standard deviation, mean squared error, variance, minimum, maximum, skewness, kurtosis, mean of auto-correlation, mean of auto-variance, standard deviation of auto-correlation, standard deviation of auto-variance, etc. Duration of gait cycle, or step length and its duration, magnitude of sensor data, and angle formed by the sensor data were used by a few other teams. T2, T3, T5, T6, T9, and T10 used sliding windowing technique in some of their algorithms over the data with some % of overlap (e.g., 50%, 75%) of the windows. Table 4 summarizes the preprocessing steps and features, which were employed by the different approaches for gender (G) classification and age (A) estimation.

T1 used Fourier transform on input training data. After the Fourier transformation of the first 100 accelerometer data, they took half of the result for calculation. T2 applied sliding window with 50% overlap for segmenting the raw data. We extracted 12 statistical features separately from both accelerometer and gyroscope.

T3 normalized the length of a gait sequence before processing them through CNN. Accelerometer data were fed into a CNN and gyroscope data were fed into another CNN, then they concatenated them. They divided each sequence into subsequences of 100 samples with an overlap of 75% between sub-sequences to collect an entire gait cycle and increase the number of samples. They have used the raw data, and allowed the model learning combined features automatically. This work is based on the work in [58]. The prediction of ‘age-class’, ‘age-regression’ and ‘gender’ are carried out using the network.

There were no preprocessing steps for T4. They introduced the gender prediction algorithm based on three Long Short-Term Memory (LSTM) layers. As input, the 6D values from the accelerometer and gyroscope were fed in parallel to the 1st LSTM layer. The activation layer using ReLU activation function was followed by the last LSTM layer. A hidden layer was used before passing the feature vector for classification. For the age estimation, they introduced a binary age prediction tree, similar to a binary search tree. At the bottom, age groups are arranged as 2–10, 11–18, 19–25, 26–35 under 2–25 branch; and 36–42, 43–50, 51–60, and 60+ under the other child of the tree. Based on this structure, they split the entire training dataset, and built the ConvLSTM model for each subset.

However, T5 applied a segmentation window with window size = 100 to sample the sequences from the raw sequences. With experiments, they allowed 50% overlap, similar to the T2. T5 applied Z-score (standard score) standardization after segmentation for each feature. In the Alg.2 and Alg.3, they removed some series of data that have low variances of both angular velocity and acceleration based on some threshold values. In Alg.1 and Alg.2, they used a deep neural network architecture, inspired by the network in [59], where bidirectional LSTM (BLSTM) were explored, instead of [59]’s simple LSTM model. For age estimation, they categorized age into eight classes.

T6 extracted features that are invariant to translation and rotation by converting the accelerometer and gyroscope data into magnitude and angle, which were formed by the sensor data in the 3D space. Accelerometer and gyroscope data were processed with a representation of angular velocity and magnitude time series, called Angle Embedded Gait Dynamic Image (AE-GDI) [60], which is a 2D representation of gait dynamics and it is invariant to rotation and translation. Note that the encoding strategy of AE-GDI is similar to the encoding scheme of Gait Dynamic Image (GDI) [55].

T7 submitted seven solutions for gender classification and another seven approaches for age estimations. For example, data were separated based on sensor position and activity for the model training and testing stages by T7 in the Alg.1. More than 100 high-level features were derived from the accelerometer traces (and gyroscope data were ignored). T7 used the sensor orientation-invariant method by [55] in Alg.2. Gait sequences are presented by Gait Dynamic Image (GDI) [55], which can be extracted for both accelerometer and gyroscope. For each of these signals, Short Time Fourier Transform was used to extract a frequency representation. Eigen coefficients per state were extracted. On the other hand, the other solutions exploited two filtering steps: first, they deleted the sequences belonging to users who were absent from the labeled list of subjects provided by the GAG19 organizer; and secondly, they excluded sequences shorter than a specific period, that was taken empirically.

The Alg.4 and Alg.5 of T7 were the same except some variations of hyper-parameters. Apart from the rotation invariant strategies, taken in Alg.4, Alg.5 and Alg.6, the entire process has explored Temporal Convolution Networks (TCNs) [61] as a feature extractor along with some fully connected layers. However, Alg.4 to Alg.6 have considered a few steps. In Alg.4 and Alg.5, vertical and horizontal acceleration components were computed from accelerometer data based on [62]. Then they computed the corresponding velocities by integrating the linear accelerations and their corresponding jerks. Note that they did not incorporate any gyroscope data. No preprocessing steps were done by Alg.3 and they consider TCN [61] directly. In Alg.6, Angle Embedded Gait Dynamic Image (AE-GDI) [60] representation was extracted. The AE-GDI provides orientation invariant representation of gait based on Gait Dynamic Image (GDI) [55]. The computed AE-GDIs were fed to the deep neural network. In their last solution (Alg.7 of T7), an ensemble method was explored, which was a linear combination of the previous methods used for their above solutions.

T8 introduced three solutions where the preprocessing steps were the same. The slope walks were excluded from analysis in all of their solutions, since the test data is from walks on almost flat ground. PCA rotation matrix was computed for the accelerometer data. Both the gyroscope and accelerometer data were rotated using this PCA rotation matrix. Then they removed the gravity from the first principal component of the rotated accelerometer data. They detected any overlapping step cycles based on the accelerometer data. The duration of individual gait cycles (as the number of samples) was computed to create the feature vector. They resampled the accelerometer and gyroscope data corresponding to each gait cycle to 102 samples by their approaches. Therefore, the feature vector was created based on the duration of the gait cycle (as 1 value), rotated 3D accelerometer data (as 3 × 102 data), and rotated 3D gyroscope data (3 × 102 values).

T9 submitted four proposals. Alg.1 for age and gender, defined the length of the sliding window as 90 points per axis, with a 50% overlap for subsequent sliding windows. They made sure that each subject generated at least one sliding window. 27 statistical features were computed from these data as: mean of each axis (6 features); standard deviation of each axis (six features); root mean square of each axis (six features); zero-crossing rate (total number of times the signal changed from less than 0 to above 0, or vice versa per axis) (six features); mean (AI) and variance (VI) of,

(1) $$MI\left(t\right)=\sqrt{{Ax(t)}^{2}+{Ay(t)}^{2}+{Az(t)}^{2}},$$

where (Ax,Ay,Az) are the 3D accelerometer data (two features); and normalized signal magnitude area (SMA) (one feature value) [63]. They did not consider the manually-extracted Android data of *OU-ISIR Gait Database, Inertial Sensor Dataset* [52], as it lacks the variables of gyroscope data.

In Alg.2 for both cases, they found the maximum timestamp number of the training dataset (IMUZ-center, IMUZ-left, and IMUZ-right) as T=1185. Then they converted each subject’s data into a matrix with size [1185,6]. In case of any timestamp length having less than 1185, null values were padded with zero. Hence the matrix was flattened to become a vector with the length of (1185×6=7110). So, there was almost no preprocessing prior to fed these data to a neural network.

In the Alg.3, they divided the signal into several segments based on the gait cycles. The methodology of this approach is based on [15,64]. Gait cycle segmentation was accomplished based on [64]. They computed MI(t) (Equation (1)), where (Ax,Ay,Az) are the 3D accelerometer data; and

(2) $$GI\left(t\right)=\sqrt{{Gx(t)}^{2}+{Gy(t)}^{2}+{Gz(t)}^{2}},$$

where (Gx,Gy,Gz) are the 3D gyroscope data. The divided the entire signal based on the period of GI(t). Then moving average windowing technique was done to smooth the data and normalized the data to [01]. They computed step length as the total number of steps, and step duration in seconds—from the accelerometer. They also deducted mean value of the step, standard deviation of the step, global minimum of the step, global maximum of the step, root mean square value of the step, entropy (i.e., uncertainty measure of the steps), and maximum amplitude of the frequency spectrum of the signal of the step—from both accelerometer and gyroscope. Through this process, 16 features were extracted from time and frequency domains. In their final solution as Alg.4, they combined the above three models.

T10 applied a 5-s window frame with 50% overlapping and each window frame could contain 5 s times 100 Hz (i.e., 500 samples). Every single sample contained six columns of the dataset (for triaxial accelerometer and gyroscope). They explored 12 types of time-domain statistical features such as maximum, minimum, mean, median, mean absolute deviation, skewness, standard deviation, variance, kurtosis, root mean square value, standard error of mean, and vector sum [65]. In total, they extracted 88 feature values. They computed features related to statistical as well as energy information of motion data, as the walking patterns were not only on the flat floor.

### 4.2. Explored Classifiers

Classical classifiers or deep learning-based approaches were employed for classification mainly. Table 5 enlists different methods that are explored by the algorithms. For example, T1 employed a network where activation function as ‘Relu’ and ‘softmax’ were employed, along with Adam optimizer as an optimization function. T2 also used Adam optimizer in their deep neural network architecture. T2 predicted age by using an ensemble of multiple regression models, e.g., ridge regression (linear regression with Ridge method) and Random Forest regressor. However, for gender prediction, they explored four classifiers, namely, Support Vector Machine (SVM), Random Forest with different depth parameters, K-Nearest Neighbor (K = 3) along with two different architectures of neural networks.

T3 explored different loss functions and parameters in their methods. They introduced three individual models for gender prediction and four separate models for age prediction with different hyperparameters for age-class, age-regression, and gender. Afterwards, ensemble models were computed according to the following information: for gender prediction, *mode* of the model outputs used for the three models are used; for age prediction: *mean*, *mode*, and *median* of the model outputs used for the four models are used. Through these processes, there are in total four results for gender prediction, and seven algorithms for age prediction by T3.

T5’s Alg.3 and Alg.4 applied ResNet [66]. Raw gyroscope and accelerometer data were concatenated and then 16 convolutional layers were implemented. However, in Alg.4, they applied GRU layers (Gated Recurrent Unit) in between the last 1D convolutional layer ResNet block and the dense layer. For gender classification, binary cross-entropy was used as a loss function, whereas for age estimation as regression problem—mean absolute error loss was exploited. In Alg.3 and Alg.4, for age estimation, Random Forest classifier was employed for ensemble learning in the age regression problem.

In both algorithms of T6, Bidirectional Long Short-Term Memory (BLSTM) architectures were explored with slightly varied parameters, loss functions, and summation methods. For example, Alg.1 employed Label Distribution Age Encoding (LDAE) [67] as age loss function, whereas, the 2nd algorithm exploited Deep Label Distribution Learning (DLDL) [68,69] as age loss function. They implemented two different accumulation methods: for Alg.1, test predictions were accumulated with arithmetic mean, and for Alg.2, the same was done with geometric mean.

T7 exploited AutoWEKA 2.0 [70] for several machine learning algorithms. For gender classification, they explored Random Forest, SMO (it implements the Sequential Minimal Optimization (SMO) algorithm, by John Platt, for training a support vector classifier), Attribute Selected Classifier (where dimensionality of test and training data is selected or reduced based on attribute selection process before processing on to a classifier), IBk as the K-nearest neighbor classifier, and Random Subspace (it constructs a decision tree based classifier) methods. On the other hand, for age estimation, Random Forest, SMO regression (it implements the Support Vector Machine for regression), Random Subspace, and Kstar ($K^{*}$ is an instance-based classifier) algorithms from WEKA 2.0 were used.

For age estimation, Support Vector Regressor (SVR), and for gender prediction, Support Vector Machine (SVM) are used by T7’s Alg.2. From Alg.3 to Alg.6, they conveyed the extracted features in some dense layers. Mean absolute error was minimized for age estimation, whereas, classification accuracy was maximized for gender classification. They selected a fully-connected layer with two variants, namely—a linear function to generate a continuous output for age estimation; and different non-linear activation functions to deal with the binary classification problem to estimate gender.

For each gender, T8’s Alg.1 to Alg.3 used SVM regression model with RBF kernel for age prediction. On the other hand, a binary classifier, SVM model with RBF kernel was explored for gender classification. However, the SVM models had different settings for the three algorithms. For example, for Alg.1, the SVM modeling was trained or validated on alternate gait cycles from each sample. Both training and validation sets considered all subjects and all device locations (i.e., center, right, and left). On the other hand, Alg.2 considered gait cycles from the center IMUZ only for training the SVM, and gait cycles from the left and right IMUZs for validation to model the SVM. Both training and validation sets contained each subject. Finally, in Alg.3, gait data from half of the subjects were used to train the SVM model, whereas, gait data from the remaining half of the subjects were exploited to validate the model. However, both training and validation sets had no subjects in common.

For age prediction, Alg.1 of T9 applied Random Forest to predict the possibilities of each sensor location (namely, center, left and right) per window. Then, given each location, they employed Support Vector Regression (SVR) to predict the age of each window. So, they created three SVR models and finally, by averaging these predictions, the ages were estimated. On the other hand, for gender classification, they followed the same structure but instead of SVR, they created three Support Vector Machine (SVM) models for three locations. However, if the average of three predictions was larger than 0.5, the considered it as male, and vice versa.

In Alg.2 of T9, the three datasets for three locations were trained individually using convolutional neural networks. Each layer was a fully-connected layer with ReLU as activation function. For gender prediction, the output layer was softmax and no activation function was used for age. Age was decided by averaging the three models. In Alg.3, Random Forest, ridge regressor, linear regressor, Lasso regressor, Support Vector Classifier (SVC) and XGboost classifier were explored, though the later demonstrated the best result. As Alg.4, they combined their first three algorithms. For age prediction, they took an average value. Similarly, for gender classification, they also took an average of the first three predictions. However, if the average is larger than 0.5, the prediction was marked as male, and vice versa.

T10 explored k-Nearest Neighbors, Support Vector Machine with Radial Basis Function, and Random Forest classifiers for gender classification. To estimate age, k-Nearest Neighbor Regression (kNNR), Support Vector Regression (SVR), and Decision Trees Regression (DTR) were employed.

## 5. Results and Analysis

In this paper, we rigorously analyzed the experimental results for age prediction and gender estimation. In the competition, the results are evaluated by mean absolute error (years) for age prediction, and percentage of mistakes or prediction errors for gender estimation, along the line with the ground-truth for both age and gender, respectively. Results are presented separated for gender estimation and age prediction. At the end, the top algorithms and the summarized results are presented.

### 5.1. Prediction Errors of Gender Estimation

**Figure 8:**
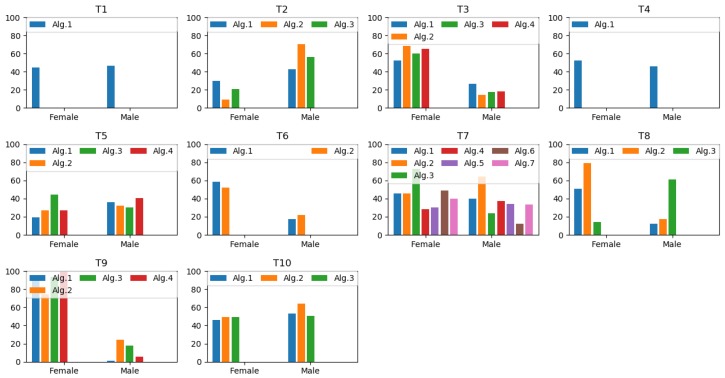
Gender prediction results for the 10 teams.

**Figure 9:**
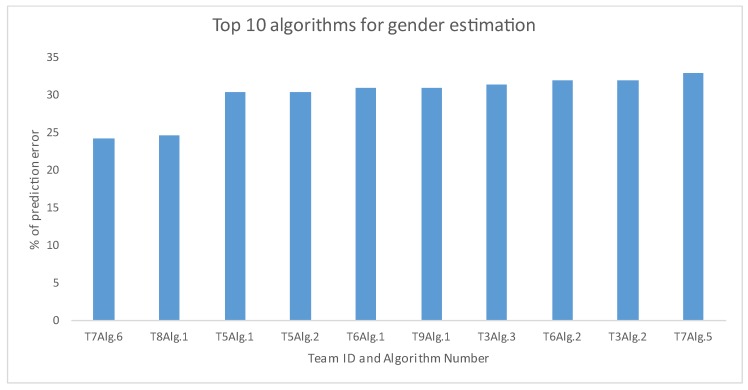
Top 10 algorithms, irrespective of any team to predict errors for gender estimation. ‘T’ stands for ‘Team’ and ‘A’ stands for ‘Algorithm’.

In this sub-section, results of prediction errors of gender estimation are illustrated in Figure 8. There 32 solutions and the prediction errors vary in both between and within the teams. For example, T1 and T4 have one solution and they showed poor results. On the other hand, T9 produced excellent prediction results for males though inferior predictions for females. Similarly, T3 and T6 also produced similar varied findings. These methods are based on deep learning-based algorithms. However, T2 demonstrated the opposite results, and they explored a conventional support vector machine as classifier. T7 presented seven solutions in different ways. Hence, they showed the most diverse prediction results within the team. Meanwhile, T10 gave the poorest prediction solutions along with T7’s Alg.2 (the worst predictions among all solutions) and T2’s Alg.2. Figure 9 demonstrates the top 10 algorithms from different teams to predict errors for gender estimation. We notice that the Alg.6 of T7 was the superior one.

**Figure 10:**
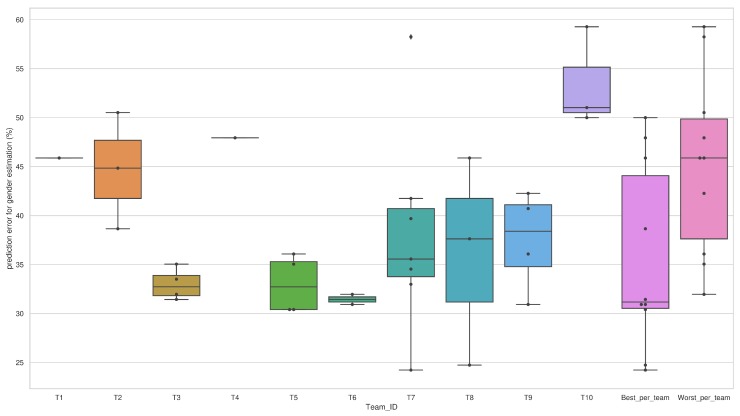
Comparison of different algorithms by teams in terms of the distribution of prediction error for gender estimation.

We can see from Figure 10, T10 has the highest median error for gender estimation. In addition, the algorithm used by this team results in the highest prediction error among all, which represents poor generalizability and poor robustness of their algorithm for this dataset. Though T7 has the lowest prediction error for the gender, we can see from the graph that their median error was higher than the median error of some other teams. The reason behind that is their highest amount of submission (seven algorithms in total). This represents that all of their algorithms were not robust. The interquartile range was maximum for T8, which represents the maximum variability in terms of performance among their submitted algorithms. In addition, the difference between the maximum and minimum error was larger for algorithms submitted by T8, which indicates wider distribution, that is, more scattered error range. In other words, we can say that their algorithms were not similar at all in terms of performance. The shortest interquartile range of T6 signifies less variability that means their submitted algorithms were mostly similar. Though the submitted algorithms of T7 performed well, one algorithm by them resulted in an error of 58.25% error, whereas all the other algorithms were very much lower than this value. This situation has been marked as an outlier (diamond shape) in the graph. In addition, we can see from the graph that T1 and T4 have submitted only one algorithm each. We have also plotted the error range for the best error rate (lowest error per team) and the worst error rate (highest error per team). These two plots with a higher interquartile range signify the variability of submitted algorithms by the ten teams.

### 5.2. Prediction Errors of Age Estimation

**Figure 11:**
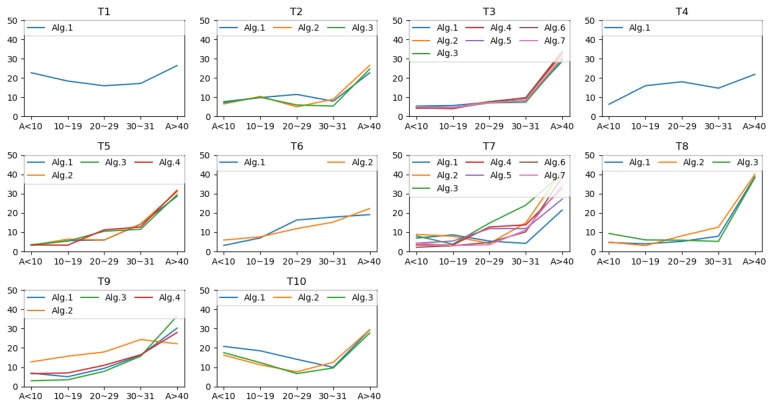
Age prediction results by age groups for the 10 teams.

**Figure 12:**
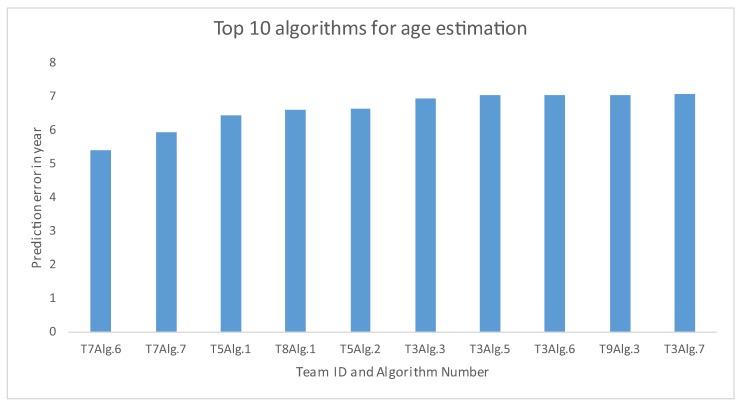
Top 10 algorithms, irrespective of any team for age prediction results by age groups. ‘T’ stands for ‘Team’ and ‘A’ stands for ‘Algorithm’.

The prediction errors of age estimation are depicted in Figure 11. Overall, a dominant trend among the 35 solutions is noticeable: the younger subjects have shown better age estimation performance than that for older age groups. Top results are found from T7, T5, and T8, while T1 and T10’s Alg.1 produced very inaccurate predictions compared to the other methods. We extracted the top ten approaches for age estimation from the 35 algorithms from 10 teams. Figure 12 shows that T7 has two algorithms that showed the best performance for age estimation.

**Figure 13:**
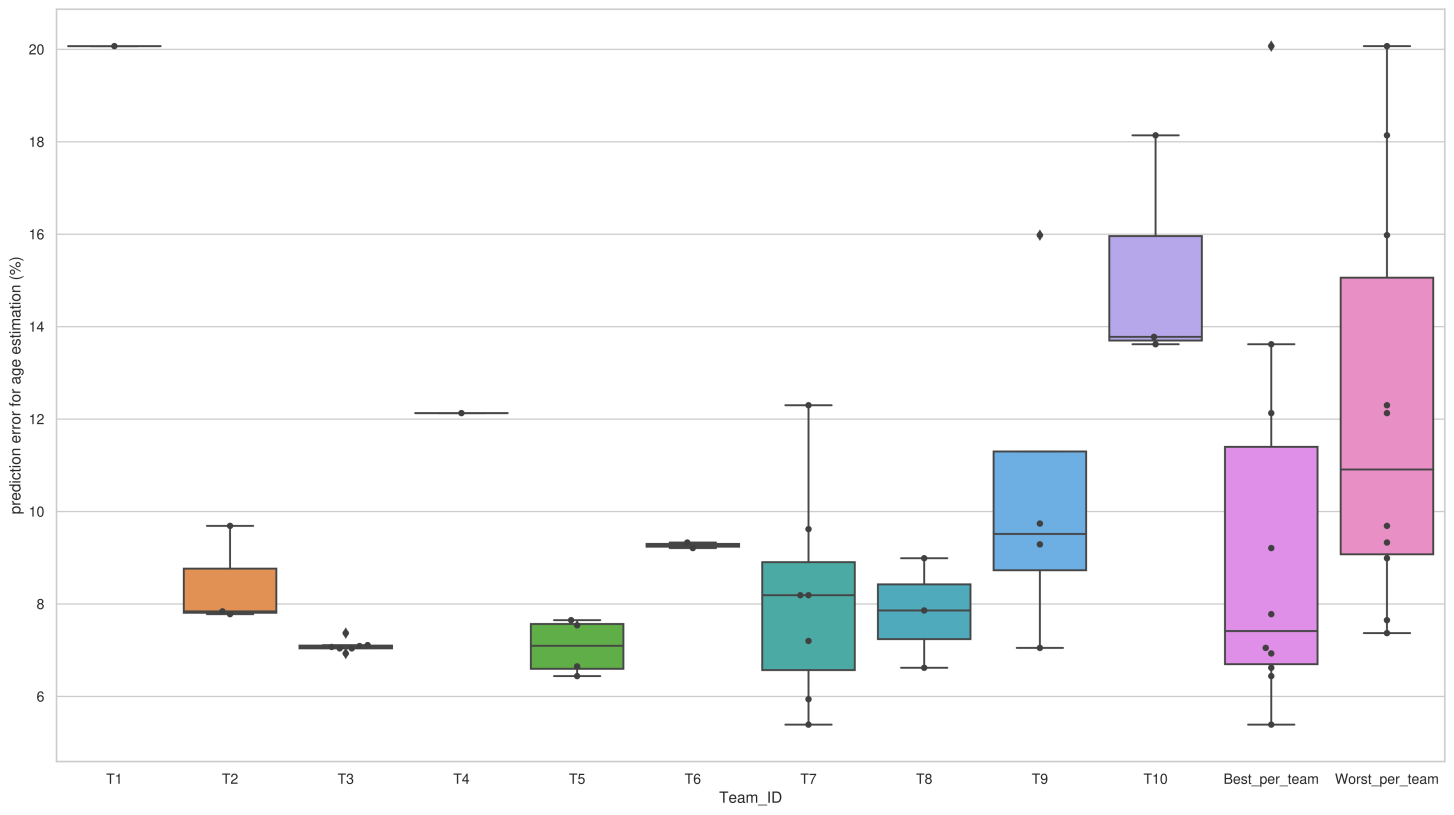
Comparison of different algorithms by teams in terms of the distribution of prediction error for age estimation.

We can see almost a similar scenario from Figure 13 like Figure 10. An interesting fact, in this case, is the presence of more outliers (outliers present for T3 and T9) than gender estimation. This means that among the submitted algorithms by T3 and T9, there were two algorithms for T3 and one for T9 which resulted in higher error than their rest of the submitted algorithms for age estimation. In this case, also the lower interquartile range of errors for T6 indicates that their submitted algorithms performed almost similarly. For T10, their median error rate was almost near their minimum error rate, which means they had mostly similar performed algorithms and they work better than the rest, but their maximum error rate was highest than the other teams. In addition, their minimum error rate was larger than the maximum error rate of some other teams, which signifies very poor generalizability and robustness of their algorithms than the rest. In addition, the higher interquartile ranges for T7, T9, and T10 signify the variability of their algorithm in terms of performance.

### 5.3. Results Summarization

Here, we summarize and compare the all solutions for all teams. We finally demonstrate the top algorithms. The overview of the gender prediction results is presented in Table 6, and the comparison of predicted errors for age prediction is demonstrated in Table 7. As mentioned above, except a few solutions, most of the algorithms did not consider the location inconsistency or sensor orientation invariance issue. Therefore, methods that did not make their methods invariant to location or orientation, achieved inferior results.

As we can notice on gender prediction from the Table 6, the top result was achieved by the Alg.6 of T7 (prediction error of 24.23%). The next best result was accomplished by the Alg.1 of T8 (prediction error of 24.74%). Both of them handled the sensor orientation or location inconsistency constraint (as shown in Table 3). The top solution (Alg.6 of T7) explored deep learning-based solution, whereas, the later solution (Alg.1 of T8) implemented Support Vector Machine (which is a very widely-explored machine learning technique). However, the third-best solutions are from Alg.1 and Alg.2 of T5 (prediction error of 30.41%). They did not consider any remedy for sensor inconsistency and yet achieved better results. Note that their results are far from the best two solutions. Other results are lower, in twice more errors than the top-ranked solutions.

For age estimation, the Alg.6 of T7 achieved the best result (mean absolute error of 5.39 years). Note that this algorithm also achieved the best result in gender prediction. Moreover, the Alg.7 of T7 also accomplished the second-best solution for age estimation among the all teams (men absolute error of 5.94 years). Alg.7 has ensembled the prior solutions of T7, therefore, this excellent result may be responsible for the best solution of Alg.6 of this team. Note that T7’s solutions jumped from the best 5.39 years to even 12.30 years, where the later is a poor result. The next best result was achieved by the Alg.1 of T5, having the mean absolute error of 6.44 years. Note that this algorithm did not consider location- or orientation-invariant solution. T5’s Alg.2 also achieved superior results (mean absolute error of 6.65 years). Alg.1 of T8 also achieved top results of the mean absolute error of 6.62 years. The worst results came from T1 and T10. Overall, the Alg.6 of T7 performed the best in both age and gender estimation. This solution computed the Angle Embedded Gait Dynamic Image (AE-GDI) representation to make it orientation-invariant, and then employed 2D convolutions and pooling layers, few fully-connected layers, and a task-specific output layer. The chance-level errors are 32.47% and 11.34 years for gender and age prediction, respectively. The error is computed when predicted results are constantly the average of the ground-truth label (age or gender) of training data. Some algorithms considered random guess. Note that some solutions demonstrated poor results. The main reason is that the dataset was challenging. The train and test datasets were captured at different sensor-orientation and sensor-translation. In the submitted algorithms, no solution handled the sensor-translation issue, whereas, some methods addressed the aspect of sensor-orientation (as shown in Table 3). Based on the top 10 approaches on gender classification as well as age estimation, seven of them considered coordinate transformation. Deep-learning based methods led the ladder in both cases. For gender classification, out of the top ten methods, four of them explored BLTSM, two methods used TCN and another two methods explored CNN. Similarly, the top ten teams on age estimation, we can find that four approaches used CNN, two algorithms exploited BLSTM, and another two considered TCN. Statistical feature-based methods achieved average results overall.

## 6. Conclusions

Wearable sensor-based gait analysis for gender and age estimation are covered in this paper based on a global competition, arranged at *The 12th IAPR International Conference on Biometrics* (ICB), Greece, 2019. It is based on a challenging dataset, and 18 teams did the registration from fourteen countries. Finally, 10 teams submitted 32 solutions for gender estimation and 35 algorithms for age estimation. The test dataset was challenging. Though most of the teams submitted multiple solutions, only a number of them achieved reasonably good results for both gender and age estimation. Various types of preprocessing steps, feature extraction strategies, and classification approaches were explored. Deep learning-based methods provided top results compared to other methods. The best result was achieved for both age and gender estimation by exploiting the angle embedded gait dynamic image and then employing a temporal convolutional network. Multi-task BLSTM-based approach provided good results too. These outcomes demonstrate that sensor-based gait and walking patterns can be very much useful for gender and age estimation. In the future, we would like to explore few strategies based on the above analysis to achieve better results than the presented results in this paper. Based on the experiences of this challenge, we can introduce similar kinds of challenges for further research development.

## Figures and Tables

**Table 1:** Detailed information about the 18 registered teams for the competition.

Team No.	Team Name	Affiliation of the Team
T1	Nii Lab!	University of Hyogo, Japan
T2	AnythingWouldDo	National University of Singapore, Singapore
T3	VIP-AC-UMA	University of Màlaga, Spain
T4	NBL	Norwegian University of Science and Technology, Norway
T5	Three Kingdom	So-net Media Networks Corp.,
		University of Southampton, UK,
		Taiping Financial Technology Service Co., Ltd.
T6	Orange Labs	University of Technology of Troyes, France
T7	KU Leuven	imec-DistriNet and imec-COSIC, KU Leuven, Belgium
T8	USF-CSE-CVPR	University of South Florida, USA
T9	NCTU-YJ lab	National Chiao Tung University (NCTU), Taiwan
T10	Ekattor	University of Dhaka, Bangladesh
T11	NPS	Naval Postgraduate School, USA
T12	snakesoft	Shenzhen Institute of Advanced Technology, China
T13	SIATMIS	China
T14	Code Surfers	National University of Science and Technology, Pakistan
T15	JG-ait	University of Hildesheim, Germany
T16	Just Yellow	University of Hildesheim, Germany
T17	Unipi_GC	University of Pisa, Italy
T18	Anonymous	Norwegian University of Science and Technology, Norway

**Table 2:** Total number of submitted algorithms by different teams—for Gender Prediction (GP) as well as Age Prediction (AP).

Team No.	#Algorithms for GP	#Algorithms for AP
T1	1	1
T2	3	3
T3	4	7
T4	1	1
T5	4	4
T6	2	2
T7	7	7
T8	3	3
T9	4	4
T10	3	3
Total	32	35

**Table 3:** Approaches to sensor-orientation invariance, managed by the participants.

Orientation Management	Team No.
By the magnitude of raw accelerometer or gyroscope signals	T6 (for all algorithms)
By using a pair of motion vectors for accelerometer, and
rotation angle around the 3D rotation axis for gyroscope [55]	T7 (for Alg. 2,4,5,6)
By PCA-based rotation matrix	T8 (for all algorithms)
By random rotations of inputs during training	T3 (for all algorithms)

**Table 4:** Employed preprocessing and feature extraction approaches for gender (G) classification and age (Ag) prediction. ‘*’ denotes that all of their algorithms used the approach. Numbers represent the algorithm numbers (i.e., instead of ‘Alg.1’, ‘Alg.2’—we mention ‘1’, ‘2’).

Preprocessing									Feature								
Team No.	Gender (G)/Age (Ag)	Windowing	Signal Normalization	Coordinate Transformation	Low-Variance Data Removal	PCA Matrix Calculation	Gyroscope Exclusion	Z-Score Standardization	HMM-UBM	Raw Data	Fourier Transform	Gait Cycle Calculation	Gait Dynamic Image (GDI)	Angle Embedded GDI	Statistical Features Extraction	Eigen Projection Matrix	Ensemble of Previous Methods
T1	G										1						
.	Ag																
T2	G	*													*		
.	Ag	*													*		
T3	G	*	*	*						*		*					4
.	Ag	*	*	*						*		*					5–7
T4	G									1							
.	Ag									1							
T5	G	*			2–4			*									
.	Ag	*			2–4			*									3, 4
T6	G	*		*										*			
.	Ag	*		*										*			
T7	G			2–5			1		2	3	2		2	6		2	7
.	Ag			2–5			1		2	3	2		2	6		2	7
T8	G			*		*						*					
.	Ag			*		*						*					
T9	G	1, 3	3							2		3			1, 3		
.	Ag	1, 3	3							2		3			1, 3		
T10	G	*													*		
.	Ag	*													*		

**Table 5:** Classifiers for gender (G) classification and age (Ag) prediction. ‘*’ denotes that all of their algorithms used the approach. Numbers represent the algorithm numbers per team.

Team No.	Gender (G)/Age (Ag)	CNN	ConvLSTM	Bidirectional-LSTM	Conv. GRU DNN	ResNet-Based Net.	Temporal Conv. Network	Random Forest	K-Nearest Neighbor	Support Vector Machine	Support Vector Regressor	Random Subspace	XGboost Classifier	Support Vector Classifier	Sequential Minimal Optimization	KNN Regressor	Decision Tree Regressor	Ridge Regressor	Binary Age Tree	KStar	Ensemble Methods
T1	G	1																			
.	Ag	1																			
T2	G	2						*	*	*											*
.	Ag							*										2			*
T3	G	*																			4
.	Ag	*																			5–7
T4	G		1																		
.	Ag		1																1		
T5	G	1, 2		1, 2	4	3, 4		3, 4													*
.	Ag			1, 2	4	3, 4		3, 4													*
T6	G			*																	
.	Ag			*																	
T7	G	6					3–6	1	1	2		1			1						7
.	Ag	6					3–6	1			2	1			1					1	7
T8	G									*											
.	Ag										*										
T9	G	2						1,3		1											
.	Ag	2						1,3			1		3	3				3			
T10	G							3	1	2											
.	Ag										2					1	3				

**Table 6:** Comparison of predicted errors for gender estimation. Note that 8 out of 10 teams submitted multiple results using different algorithms (Alg.). ‘Best/team’ is the Best result for each team is considered. **1st** position is highlighted as **bold**; ***2nd best*** result is shown as ***bold-italic***; and *3rd best* result is marked by *italic*.

Team	% of Mistake or Prediction Errors for Gender Estimation							
	Alg.1	Alg.2	Alg.3	Alg.4	Alg.5	Alg.6	Alg.7	Best/Team
1	45.88							45.88
2	38.66	50.52	44.85					38.66
3	35.05	31.96	31.44	33.51				31.44
4	47.94							47.94
5	30.41	30.41	35.05	36.08				*30.41*
6	30.93	31.96						30.93
7	41.75	58.25	39.69	34.54	32.99	24.23	35.57	**24.23**
8	24.74	37.63	45.88					***24.74***
9	30.93	40.72	42.27	36.08				30.93
10	51.03	59.28	50.00					50.00

**Table 7:** Comparison of predicted errors as mean absolute error (year) for age estimation. Note that 8 out of 10 teams submitted multiple results using different algorithms (Alg.). ‘Best/team’ is the Best result for each team is considered. **1st** position is highlighted as **bold**; ***2nd best*** result is shown as ***bold-italic***; and *3rd best* result is marked by *italic*.

Team	Prediction Errors for Age Estimation on Various Algorithms							
	Alg.1	Alg.2	Alg.3	Alg.4	Alg.5	Alg.6	Alg.7	Best/Team
1	20.07							20.07
2	9.69	7.78	7.84					7.78
3	7.37	7.11	6.93	7.09	7.04	7.04	7.07	6.93
4	12.13							12.13
5	6.44	6.65	7.54	7.65				***6.44***
6	9.21	9.33						9.21
7	7.20	9.62	12.30	8.19	8.19	5.39	5.94	**5.39**
8	6.62	7.86	8.99					*6.62*
9	9.29	15.98	7.05	9.74				7.05
10	18.14	13.62	13.78					13.62
